# Injury to the Soft Tissues by Dentures

**Published:** 1911-06-15

**Authors:** R. Mckay


### INJURY TO THE SOFT TISSUES BY DENTURES.

BY R. MCKAY, L.R.C.P., M.R.C.S., L.D.S.

When one considers the numbers of ill-fitting dentures which are in use, it is surprising how little serious injury to the soft tissues of the mouth is seen in practice. It is possible that the majority of cases which become malignant pass direct to the general surgeon, but it would seem that the buccal mucous membrane is singularly tolerant of prolonged injury.

Exclusive of the injury sometimes seen with new dentures where the bite has been left hard, two main classes of injury may be considered, *viz.*

(1) Absorption of the alveolar processes with consequent settling of the denture.

(2) Uncleanliness and a general septic condition of the mouth and dentures.

Two cases presented themselves at the hospital last year, which well illustrate these types.

In one, direct injury resulted from the edge of a plate. The plate, an upper one, had been worn for fifteen years; odd natural teeth had been lost since, and absorption of the alveolar processes had taken place, with the result that the edge of the plate—itself thin and sharp—had cut into the soft tissues. The patient was a female over 70, and had noticed the sore place for two and a half years, but being unable to afford new teeth she put up with the discomfort as long as she was able. The ulcer was deep and clean cut, and with distinct hardening around. The mouth itself was clean.

The plate was left out, and the patient watched in view of possible malignancy. Marked improvement was seen in two weeks: the induration cleared up, and the ulcer slowly decreased in size, but a narrow fissue-like condition remained for over three months. This has now disappeared and the epithelium over the part appears quite normal. All pain ceased with the removal of the denture, but the patient is still being kept under observation on account of her age and the duration of the injury.

In the other case vulcanite dentures had been worn for fifteen years, day and night. These, although "always cleaned," were filthy and the mouth generally in a septic condition. The mucous membrane under the dentures was deeply congested, and the pathological report was "epithelium slightly hypertrophied, otherwise normal without suggestion of new growth." In the left lower and right upper canine regions were large granulating ulcers. the condition cleared up with simple cleanliness and removal of the dentures.

These cases are of interest, since it is the association of long-continued irritation with sepsis which may be the starting point of malignant growths of the jaws. In the gums the growth may start from the edge round a septic tooth and invade the bone, whereas in the hard palate the edge of the plate may start the new growth.

With the absorption of the alveolar process in the mandibular incisor region the inner edge of the denture frequently causes a marked ulceration under the tongue. From a practical point of view it should be remembered that a well recognized type of carcinoma occurs in this region called "sub-lingual" cancer. In ulceration apparently due to a denture it is well to exclude the possibility of the ulceration being malignant.

The septic condition illustrated by the second case is entirely preventible by simple cleanliness. It occurs in those who keep their dentures in the mouth day and night. Scrupulous cleanliness will not in these cases always prevent irritation. It is more marked when the surfaces in contact with the soft parts are of unpolished vulcanite, but is also seen with neglected metal cases.—*Dental Record.*

